# Hyperhomocysteinemia reduces the high-quality embryo rate in PCOS patients undergoing IVF/ICSI: clinical evidence and a preliminary exploration of mechanisms in KGN cells

**DOI:** 10.1186/s13048-026-02120-y

**Published:** 2026-05-13

**Authors:** Haofei Shen, Tianyu Jia, Xiaorong Luo, Jianxiu Zheng, Chunjie Zhang, Shan Gao, Xuehong Zhang, Junjun Huang

**Affiliations:** 1 The First Hospital of Lanzhou University, Lanzhou, Gansu 730000 China; 2 The First Clinical Medical College of Lanzhou University, Lanzhou, Gansu 730030 China; 3 The Second People‘s Hospital of Gansu Province, Lanzhou, China; 4 Gansu Provincial Maternity and Child-care Hospital, Lanzhou, China

**Keywords:** Homocysteine, Polycystic ovary syndrome, Network pharmacology, Cleavage-stage high-quality embryo rate

## Abstract

**Background:**

Homocysteine (Hcy) is a sulfur-containing intermediate metabolite during methionine metabolism. Studies indicate that individuals with polycystic ovary syndrome (PCOS) have higher circulating Hcy levels relative to unaffected populations. Hyperhomocysteinemia (HHcy) has been implicated in multisystem cytotoxicity. This study was designed to evaluate the association between Hcy levels and embryo quality and pregnancy outcomes in PCOS patients undergoing assisted reproductive technologies (ART). Mechanistic insights were explored through in vitro assays and combined network pharmacology analyses.

**Methods:**

A total of 599 patients with PCOS who were undergoing in vitro fertilization/intracytoplasmic sperm injection (IVF/ICSI) were retrospectively analyzed. Patients were stratified into three groups based on Hcy levels (low, intermediate, and high). Comparative analyses of clinical and laboratory parameters were performed across strata. Multivariable logistic regression models were developed to evaluate the independent association between Hcy levels and reproductive outcomes after adjustment for potential confounders. In vitro assays were conducted using KGN cells, a human granulosa-like tumor-derived cell line. Cellular viability after exposure to graded concentrations of Hcy was quantified using the Cell Counting Kit-8 (CCK-8) assay, while cell apoptosis was observed *via* flow cytometry. For systems-level analysis, Hcy-associated targets were retrieved from SwissTargetPrediction, TargetNet, BATMAN-TCM, and PharmMapper databases. PCOS-related targets were curated from OMIM, GeneCards, DrugBank, and DisGeNET repositories. Overlapping targets were analyzed for protein-protein interaction (PPI) network construction using the STRING platform, followed by Gene Ontology (GO) annotation and Kyoto Encyclopedia of Genes and Genomes (KEGG) pathway enrichment analyses.

**Results:**

Comparative univariate analysis showed a significant difference in the high-quality embryos among the three Hcy strata, whereas other clinical parameters did not reach statistical significance. Multivariable regression analysis identified a robust inverse association between Hcy level and high-quality embryo rate (β = -1.04, 95% CI: -1.36 to -0.72; *p* < 0.0001), which remained stable after adjustment for confounding variables, including creatinine clearance rate (β = -1.06, 95% CI: -1.40 to -0.72; *p* < 0.0001). In vitro results showed that exposure to Hcy (80–160 µM, 24 h) significantly reduced KGN cell viability and increased apoptotic rates in a concentration-dependent manner. Network pharmacology analysis identified 102 intersecting targets that may mediate Hcy-associated effects in PCOS. KEGG pathway enrichment revealed significant involvement in lipid metabolism and atherosclerosis, viral infections (i.e., hepatitis B and cytomegalovirus), the AGE-RAGE signaling pathways in diabetic complications, and the TNF signaling cascade.

**Conclusion:**

Higher Hcy levels were inversely correlated with high-quality embryo formation in PCOS patients, with each increase in Hcy associated with a measurable decline (1.4%) in embryo quality. Experimental validation indicated that Hcy exerts cytotoxic and pro-apoptotic effects on KGN cells. Combined network pharmacology further suggested that Hcy may influence PCOS. These results support a contributory role of HHcy in impaired PCOS and provide a preliminary mechanistic understanding for future investigation.

## Introduction

Homocysteine (Hcy), a non-protein, sulfhydryl-containing amino acid involved in one-carbon metabolism and generated during methionine demethylation, has cytotoxic properties and contributes to multisystem tissue damage [1]. Higher Hcy levels may arise from aging, genetic factors, deficiencies of folate and B vitamins, excessive methionine intake, hypothyroidism, chronic alcohol consumption, renal impairment, and malignancies (breast, ovarian, and pancreatic cancers) [2]. Hyperhomocysteinemia (HHcy) is defined by increased plasma Hcy levels, with normal levels ranging from 5 to 15 µmol/L. Based on severity, HHcy is categorized as mild (15–30 µmol/L), moderate (30–100 µmol/L), and severe (> 100 µmol/L) [3]. In female reproduction, HHcy induces oxidative stress, promotes platelet activation, disrupts hemodynamics, and stimulates vascular smooth muscle cell proliferation. It activates the unfolded protein response (UPR), leading to endothelial apoptosis through excessive generation of reactive oxygen species (ROS). These changes impair endometrial perfusion and vascular integrity, contributing to obstetric complications, i.e., recurrent miscarriage, gestational diabetes mellitus, hypertensive disorders of pregnancy, intrauterine growth restriction, placental abruption, and preterm birth, ultimately resulting in adverse pregnancy outcomes [4, 5]. Higher Hcy levels are also associated with reduced fertilization rates, impaired embryo quality, decreased implantation and clinical pregnancy rates, and increased risk of miscarriage [6]. HHcy disrupts folliculogenesis, oocyte competence, embryo development, and endometrial receptivity, thus increasing the possibility of implantation failure and pregnancy-related complications. In assisted reproductive technologies (ART), HHcy and variants of 5,10-methylenetetrahydrofolate reductase (MTHFR) are associated with reduced oocyte yield and compromised embryo quality; however, folate and B vitamin supplementation may partially ameliorate these effects [7]. Proteomic analyses have shown that HHcy induces mitochondrial dysfunction and downregulates zona pellucida proteins (ZP1, ZP2, and ZP3) in oocytes. Ultrastructural evaluation by transmission electron microscopy (TEM) has revealed aberrant zona pellucida formation and microvillar architecture in oocytes from HHcy models. Moreover, in vitro fertilization studies have shown reduced rates of two-cell embryo formation under HHcy conditions, indicating impaired early embryonic development and a diminished reproductive lifespan due to compromised follicular development and oocyte quality [8].

Polycystic ovary syndrome (PCOS) is a multifactorial disease that affects the endocrine, reproductive, metabolic, and cardiovascular systems. It is clinically characterized by hyperandrogenism, ovulatory dysfunction (anovulation or oligo-ovulation), infertility, and recurrent pregnancy loss [9]. Based on the Rotterdam criteria established in 2003 by the European Society of Human Reproduction and Embryology and the American Society for Reproductive Medicine, the global prevalence of PCOS ranges from 6 to 21%, with reported rates of 5.6–11.2% in China [10, 11]. PCOS is a major cause of female infertility, induced by ovulatory dysfunction, impaired oocyte development, dysregulated gonadotropin secretion, hyperandrogenism, and abnormalities in ovarian growth factors and their binding proteins [12]. Clinical evidence indicates that patients with PCOS have considerably higher Hcy levels than those without PCOS [13]. Moreover, PCOS is strongly associated with metabolic disturbances, including dysregulated glucose and lipid metabolism, which increase the risk of cardiovascular disease [14]. HHcy has been identified as an independent risk factor for cardiovascular disease and is involved in the long-term cardiovascular complications of PCOS. It is also correlated with key clinical features of PCOS, including obesity, dyslipidemia, insulin resistance, hyperandrogenism, and ovulatory dysfunction [15].

A well-regulated microenvironment is essential for optimal oocyte maturation and embryo development [16]. Follicular fluid and ovarian granulosa cells provide oocytes with critical nutrients, hormones, and enzymatic support, thus establishing the preovulatory niche required for follicular growth and ovulation [17]. Changes in follicular fluid composition can significantly affect oocyte maturation and embryogenesis [18]. The KGN cell line, a widely used human granulosa cell model in reproductive biology, retains responsiveness to follicle-stimulating hormone (FSH) and steroidogenic activity, and is extensively used to investigate granulosa cell function and ovarian pathophysiology [19–22].

Current research has predominantly focused on the association between Hcy and PCOS; however, the underlying molecular targets linking these factors remain poorly characterized, particularly using integrative approaches such as network pharmacology. Moreover, the direct effects of Hcy on ovarian granulosa cell function have not been comprehensively elucidated. Therefore, this study evaluates the effects of different serum Hcy levels on early embryonic development in patients with PCOS undergoing ART. In vitro experiments are conducted to investigate the effects of varying Hcy levels on the viability and autophagic activity of KGN cells, identifying potential molecular targets linking Hcy and PCOS.

## Materials and methods

### Research subjects and eligibility criteria

This study enrolled women who sought infertility treatment at the First Hospital of Lanzhou University between April 2022 and July 2023. The study protocol was approved by the Ethics Committee of Lanzhou University First Hospital (Approval No. LDYYSZLLKH2022-03). All participants provided written informed consent before enrollment.

Participants were included based on the Rotterdam diagnostic criteria for PCOS. PCOS was diagnosed when at least two of the following three criteria were present: (1) oligo-ovulation or anovulation, clinically manifested as menstrual irregularity; (2) clinical and/or biochemical hyperandrogenism, including hirsutism, severe acne, androgenic alopecia, or higher serum androgen levels; and (3) polycystic ovarian morphology (PCOM) detected by ultrasound.

**Fig. 1:**
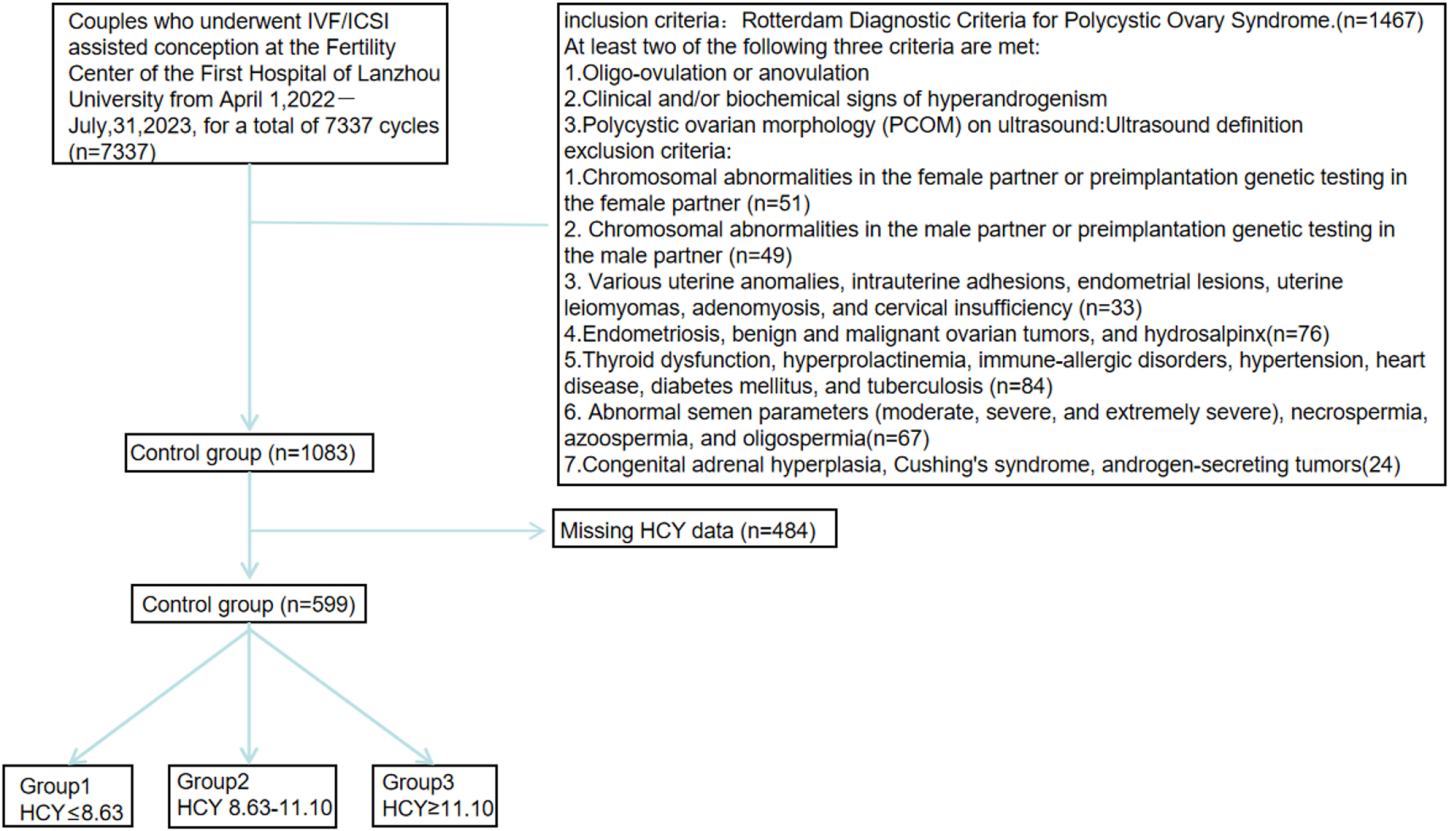
Flowchart of data exclusion and inclusion for the study

The exclusion criteria were as follows: (1) application of preimplantation genetic testing (PGT) due to heritable pathogenic variants in either female or male partners; (2) uterine abnormalities, i.e., congenital malformations, intrauterine adhesions, endometrial lesions, uterine leiomyomas, adenomyosis, or cervical insufficiency; (3) endometriosis, ovarian tumors (benign or malignant), or hydrosalpinx; (4) systemic conditions such as thyroid dysfunction, hyperprolactinemia, immune-allergic disorders, hypertension, cardiovascular disease, diabetes mellitus, or tuberculosis; (5) abnormal semen parameters, including moderate to severe oligospermia, azoospermia, or necrospermia; and (6) endocrine disorders (congenital adrenal hyperplasia, Cushing’s syndrome, or androgen-secreting tumors). A detailed patient selection flowchart is presented in Fig. 1.

Based on serum Hcy levels, patients were stratified into three groups (low, medium, and high) using tertile distribution.

### ART progress and embryo evaluation

All patients were managed at the Reproductive Medicine Center. Demographic data, including age and body mass index (BMI), were recorded, and medical histories were documented in the electronic medical record system. At enrollment, venous blood samples were collected for the assessment of complete blood count, anti-Müllerian hormone (AMH), homocysteine (Hcy), cancer antigen 125 (CA-125), and thyroid-stimulating hormone (TSH), with all analyses performed in the institutional laboratory. Transvaginal ultrasonography was conducted on menstrual cycle days 2–3 during the early follicular phase to evaluate uterine and ovarian morphology, including antral follicle count (AFC) and pelvic structures.

Oocyte retrieval procedures were performed by three senior physicians. Fertilization (IVF/ICSI), embryo culture, and morphological analysis were performed by two experienced embryologists in accordance with standardized protocols. The initial gonadotropin (Gn) dose was determined based on age, AMH, BMI, and AFC. In the long GnRH agonist protocol, 3.75 mg of agonist was administered intramuscularly during the mid-luteal phase of the preceding cycle. Gonadotropin stimulation was initiated after confirmation of pituitary downregulation (endometrial thickness ≤ 5 mm, serum estradiol ≤ 50 pg/mL, and follicular diameter ≈ 5 mm).

Ovulation triggering was performed when at least one follicle reached ≥ 18 mm in diameter, with hormone levels guiding clinical decisions. The trigger medication type, dosage, and timing were selected based on the specific stimulation protocol, follicle number, and serum hormone levels. Before oocyte retrieval, the vagina was irrigated 2–3 times. Transvaginal ultrasound-guided follicular aspiration was performed 36 h post-trigger administration. All embryos were cultured in G-1™ PLUS medium (Vitrolife, Sweden) within a tri-gas incubator maintained at 37.0 ± 0.5 °C with 6% CO₂ and 5% O₂ in a humidified atmosphere.

Pronuclear (PN) stage evaluation was performed 16–18 h post-fertilization using phase-contrast microscopy by experienced embryologists. Normal fertilization was defined by the presence of two distinct pronuclei (2PN) in the cytoplasm. Cleavage-stage embryos were graded according to the Istanbul consensus criteria. Grades I and II were classified as high-quality embryos: Grade I: Blastomeres of equal size with regular morphology, clear homogeneous cytoplasm, and < 10% cytoplasmic fragmentation, Grade II: Blastomeres of slightly unequal size with 10–20% cytoplasmic fragmentation, Grade III: Blastomeres of unequal size with 21–50% cytoplasmic fragmentation, potentially with cytoplasmic vacuolation, Grade IV: Severely irregular blastomeres with > 50% fragmentation, frequently showing prominent cytoplasmic vacuoles or granulation.

In this study, embryo transfer was performed using Grade I, Grade II, and/or Grade III embryos according to patient-specific clinical considerations. The following reproductive outcome parameters were calculated: MII oocyte rate = (number of metaphase II oocytes / total number of retrieved oocytes) × 100%; 2PN fertilization rate = (number of 2PN zygotes / total number of inseminated or injected oocytes) × 100%; High-quality embryo rate = (number of Grade I + Grade II embryos/number of 2PN zygotes) × 100%; Clinical pregnancy rate = (number of clinical pregnancy cycles/number of embryo transfer cycles) × 100% Clinical pregnancy was defined as ultrasonographic visualization of at least one intrauterine gestational sac with or without fetal cardiac activity, excluding ectopic and biochemical pregnancies diagnosed solely by serum β-hCG elevation without sonographic confirmation.

### Cell viability assay

Cell viability was observed using the CCK-8 assay (Coolaber, Beijing, China). Experimental groups included a blank control (medium only), a negative control (KGN cells without Hcy), and treatment groups exposed to Hcy at increasing concentrations (5, 10, 20, 40, 80, and 160 µM). Briefly, KGN cells (5 × 10³ cells/well) were seeded in 96-well plates and incubated for 24 h. The medium was replaced with Hcy-containing medium, followed by another 24-h incubation. Thereafter, 10 µL of CCK-8 reagent and 90 µL of medium were added to each well, and plates were incubated for 3 h at 37 °C. Absorbance was measured at 450 nm using a microplate reader (TECAN, Switzerland). Cell viability was calculated as: Cell viability (%) = [(OD_treatment - OD_blank) / (OD_control - OD_blank)] × 100. All experiments were independently performed in triplicate.

### Apoptosis detection

Apoptosis was examined using an Annexin V-FITC/PI apoptosis detection kit (MultiSciences, Hangzhou, China). Annexin V (or Annexin A5) is a member of the intracellular annexin protein family. The assay principle is based on the high-affinity, calcium-dependent binding of Annexin V to phosphatidylserine (PS). In viable cells, PS is asymmetrically distributed to the inner leaflet of the plasma membrane; however, during early apoptosis, membrane phospholipid asymmetry is disrupted, resulting in PS externalization to the outer leaflet of the plasma membrane. Fluorescently conjugated Annexin V specifically binds to externalized PS, thus identifying apoptotic cells. For this assay, KGN cells were treated with Hcy at concentrations of 0, 20, 40, and 80 µM. Cells (1 × 10⁶ − 3 × 10⁶) were collected by centrifugation, washed twice with pre-cooled PBS, and resuspended in apoptosis-positive control solution (500 µL), then incubated on ice for 30 min. After washing with ice-cold PBS, cell pellets were resuspended in an appropriate volume of pre-chilled 1X Binding Buffer. An equivalent number of untreated, viable cells was added as a negative control. The cell suspension volume was adjusted to 1.5 mL with 1X Binding Buffer and divided equally into three aliquots: (1) unstained blank control, (2) Annexin V-FITC single-stain control, and (3) PI single-stain control. For single-stain controls, 5 µL of Annexin V-FITC or 10 µL of PI was added, respectively, followed by incubation at room temperature (25 °C) in the dark for 5 min.

Flow cytometry was performed using a flow cytometer (Beckman Coulter, Cytoflex). Instrument voltage settings for forward scatter (FSC), side scatter (SSC), and fluorescence channels were optimized using the blank control. Fluorescence compensation was calibrated using the single-stain control samples. Annexin V-FITC fluorescence was detected through the FITC channel (excitation λ = 488 nm; emission λ = 530 nm), and PI fluorescence was detected through the PE/PI channel (excitation λ = 535 nm; emission λ = 615 nm. For microscopic visualization, stained cells were transferred onto glass slides and coverslipped. Before observation, cells could be fixed with 2% formaldehyde; however, Annexin V-FITC staining must be performed before fixation to preserve membrane integrity. Fluorescence imaging was performed using an inverted fluorescence microscope equipped with FITC and rhodamine filter sets. Apoptotic cells showed a characteristic green fluorescent ring at the plasma membrane (Annexin V binding), while cells with compromised membrane integrity showed red nuclear fluorescence (PI staining) in addition to peripheral green fluorescence. All experiments were independently repeated at least three times.

### Identification of Hcy targets

To predict potential molecular targets of Hcy, a multi-database approach was used, drawing on four publicly accessible online target prediction platforms: SwissTargetPrediction (http://www.swisstargetprediction.ch/) [23], TargetNet (http://targetnet.scbdd.com/home/index/) [24], BATMAN-TCM (http://bionet.ncpsb.org/batman-tcm/) [25], and PharmMapper (http://www.lilab-ecust.cn/pharmmapper) [26]. Target prediction searches were restricted to *Homo sapiens*. Retrieved gene identifiers and protein names were standardized and validated using the UniProt database (http://www.uniprot.org/) [27]. Bioinformatics analyses were conducted using publicly available, quality-controlled databases derived from validated scientific datasets.

### Identify targets for PCOS

Molecular targets associated with PCOS were retrieved from four complementary disease-gene databases: OMIM (http://www.omim.org) [28], GeneCards (https://www.genecards.org/) [29], DrugBank (https://go.drugbank.com/.) [30], and DisGeNET databases (https://www.disgenet.org/) [31], using “polycystic ovary syndrome” as the primary search query. For the GeneCards database, which assigns relevance scores that reflect the strength of gene-disease associations, only targets with a relevance score exceeding the median were retained to enhance specificity and reduce false-positive associations [32]. Target lists retrieved from all four databases were consolidated, and duplicate entries were removed to generate a comprehensive, non-redundant compendium of PCOS-associated molecular targets. The bioinformatics analyses conducted in this study are based on standard, publicly available genomic or proteomic databases. The data in these databases are derived from scientific projects that have undergone rigorous quality control and ethical review, serving as recognized, authoritative resources in the field, rather than on unverified “personal sequencing datasets.”

### Hcy‑PCOS PPI network

Venn diagram analysis was performed to identify overlapping molecular targets between Hcy and PCOS using the online platform at (https://bioinfogp.cnb.csic.es/tools/venny/). These intersecting targets were used to construct a PPI network from the STRING database (V11.5), accessible at https://string-db.org/ [31]. The species parameter was set to *Homo sapiens*, and the minimum required interaction score was defined as high confidence (> 0.7). The PPI and component-target gene–disease (CTD) networks were visualized using Cytoscape v3.9.1. The CentiScaPe plugin was used to calculate degree centrality (DC), closeness centrality (CC), and betweenness centrality (BC). Nodes showing DC, CC, and BC values above their respective mean thresholds were identified as core genes.

### Statistical analysis

Statistical analysis was conducted using SPSS v26.0 and Empower Stats. Normally distributed data were expressed as mean ± standard deviation. Independent sample *t*-tests or Mann-Whitney *U* tests were used as appropriate. Categorical variables were analyzed using chi-square tests. Hcy levels were categorized into tertiles, and intergroup comparisons were performed using analysis of variance (ANOVA) for continuous variables and chi-square tests for categorical variables. Logistic regression analysis was conducted to evaluate the association between Hcy levels and clinical outcomes, adjusting for potential confounders. Curve-fitting analysis was performed to assess the relationship between serum Hcy levels and high-quality embryo rates.

## Results

### Cycle selection process

The workflow for patient selection is illustrated in Fig. 1. Initially, 7,337 cycles were screened, including cases with potential confounding variables. After applying predefined inclusion and exclusion criteria, 1,083 patients were considered eligible for analysis. Among the remaining cohort, 484 cases were excluded due to unavailable Hcy measurements. The final analytical population was then categorized into three groups based on serum Hcy levels using a trichotomous stratification approach (Fig. 1).

### Baseline characteristics of participants

Univariate analysis revealed statistically significant differences in high-quality embryo rates among the three Hcy groups. Aside from Hcy levels and related metabolic parameters (e.g., triglycerides), which were used for stratification, no statistically significant differences were observed among the groups in key demographic and clinical baseline characteristics, including age, body mass index (BMI), and duration of infertility (Table 1).

**Table 1:** Baseline characteristics of participants

GROUP	Group 1	Group 2	Group 3	*P*-value
number	199	200	200	
Hcy(umol/L)	≤ 8.63	8.63–11.10	≥ 11.10	< 0.001
AMH(ng/mL)	7.04 ± 3.59	7.48 ± 4.08	7.51 ± 4.34	0.430
TSH(mIU/mL)	3.64 ± 2.56	3.64 ± 2.92	3.27 ± 1.93	0.232
E2(pg/mL)	37.04 ± 21.78	39.48 ± 28.53	39.59 ± 27.86	0.549
PRL(ng/mL)	22.59 ± 11.35	21.36 ± 11.71	22.35 ± 12.27	0.542
fasting plasma glucose(mmol/L)	4.84 ± 0.54	5.01 ± 1.65	4.85 ± 0.80	0.220
total cholesterol(mmol/L)	4.32 ± 0.80	4.29 ± 0.74	4.23 ± 0.78	0.552
triglyceride(mmol/L)	1.43 ± 1.07	1.46 ± 0.98	1.19 ± 0.64	0.004
high density lipoprotein(mmol/L)	1.33 ± 0.29	1.32 ± 0.28	1.38 ± 0.29	0.068
low density lipoprotein(mmol/L)	2.47 ± 0.63	2.41 ± 0.63	2.46 ± 0.67	0.683
duration of infertility (year)	3.06 ± 1.89	3.54 ± 2.26	3.40 ± 2.13	0.068
body mass index(kg/m^2^)	24.26 ± 2.86	23.89 ± 3.20	24.65 ± 3.28	0.127
antral follicle count	18.17 ± 8.08	20.39 ± 8.87	20.58 ± 10.29	0.014
menstrual cycle(day)	33.69 ± 10.87	34.43 ± 13.24	36.88 ± 16.93	0.057
duration of ovarian stimulation(day)	10.40 ± 1.91	10.20 ± 1.71	10.21 ± 2.04	0.512
Cumulative gonadotropin dose (IU)	2337.50 ± 799.32	2304.44 ± 768.20	2277.81 ± 813.95	0.754
endometrial thickness of HCG day(mm)	10.94 ± 2.83	10.83 ± 2.67	10.91 ± 2.73	0.371
MII rate(%)	87.87 ± 15.78	88.63 ± 14.44	88.47 ± 14.93	0.868
2PN rate(%)	64.11 ± 19.75	65.72 ± 20.56	63.92 ± 21.24	0.628
high quality embryos rate(%)	59.35 ± 21.20	54.71 ± 19.77	46.97 ± 22.50	< 0.001
type of infertility(%)				0.369
primary infertility	61.3	54.5	56.5	
secondary infertility	38.7	45.5	43.5	
fertilization method(%)				0.978
IVF	77.9	77.5	77.0	
ICSI	22.1	22.5	23.0	
pregnancy(%)				0.575
yes	44.2	40.0	39.5	
no	55.8	60.0	60.5	

### Multivariate analysis of factors associated with high-quality embryo rate

Multivariate regression analysis revealed a significant negative association between serum Hcy levels and the high-quality embryo rate (β = -1.04, 95% confidence interval [CI]: -1.36 to -0.72; *p* < 0.0001). This relationship remained robust after adjustment for potential confounders, i.e., creatinine clearance rate, female age, anti-Müllerian hormone (AMH), antral follicle count (AFC), thyroid-stimulating hormone (TSH), total cholesterol (TC), triglycerides (TG), and endometrial thickness (β = -1.06, 95% CI: -1.40 to -0.72; *p* < 0.0001) (Table 2). No statistically significant differences in clinical pregnancy rates were observed among the Hcy-level groups. However, higher Hcy levels were inversely associated with the cleavage-stage high-quality embryo rate, with each 1 µmol/L increase in Hcy corresponding to an approximate 1.4% reduction in this parameter.

**Table 2:** Multivariate analysis of factors associated with high-quality embryo rate

	Non-adjusted	Adjusted
HCY	-1.04 (-1.36, -0.72) < 0.0001	-1.06 (-1.40, -0.72) < 0.0001

Adjusted: female age; AMH; TSH; FSH; LH; E2; PRL; fasting plasma glucose; total cholesterol; triglyceride; high density lipoprotein; lowdensity lipoprotein; duration of infertility; body mass index; antral follicle count; menstrual cycle; endometral thickness of HCG day

### Curve modeling of the association between serum Hcy and high-quality embryo rate

**Fig. 2:**
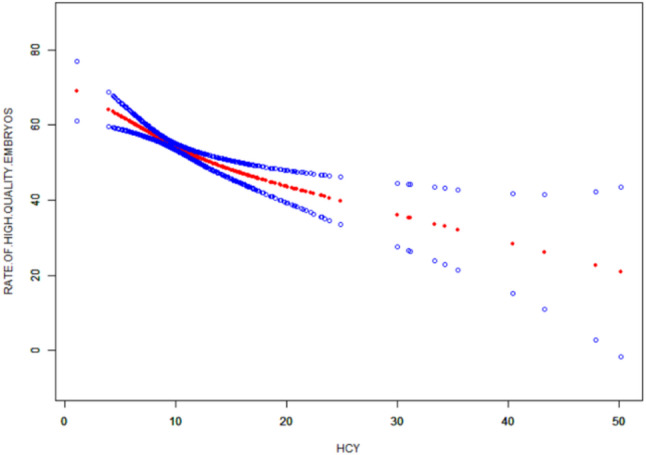
Curve modeling of the association between serum Hcy and high-quality embryo rate. (The red curve represents the curve fitting of the high-quality embryo rate, and the blue curve represents the 95% confidence interval)

Curve-fitting analysis was performed to further characterize the relationship between serum Hcy levels and the high-quality embryo rate (Fig. 2). The results showed a progressive decline in the high-quality embryo rate as serum Hcy levels increased, indicating a dose-dependent negative association.

### Effect of Hcy on cell viability

**Fig. 3:**
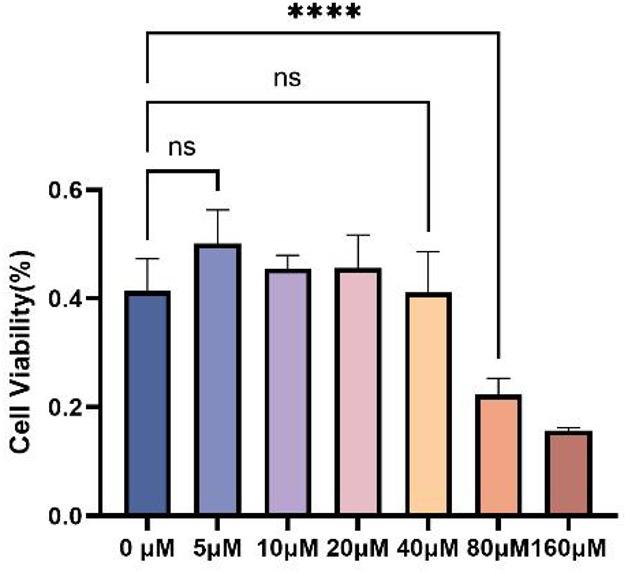
The effect of Hcy on the activity of KGN cells. (“ns” indicates no statistically significant difference; “****” denotes a *P*-value < 0.0001)

We found treating with 80 µM Hcy for 24 h significantly decreased cell viability (Fig. 3).

### Hcy-induced apoptosis in KGN cells

**Fig. 4:**
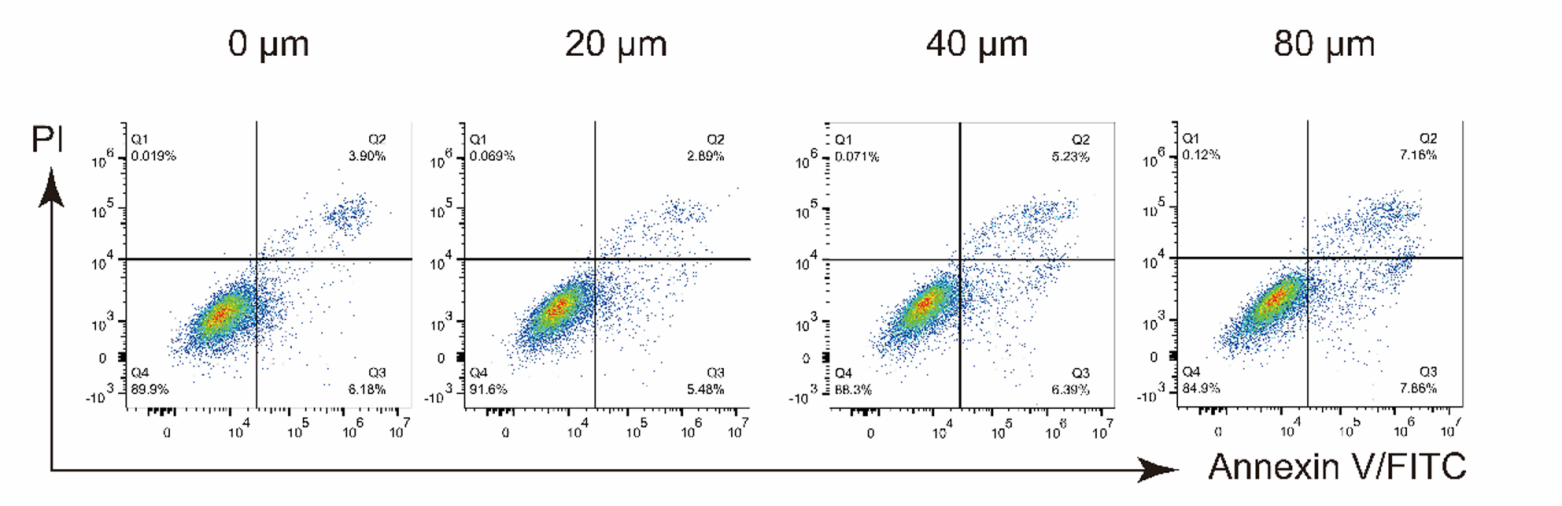
The effect of Hcy exposure in KGN cells

To elucidate the mechanism underlying KGN cell death, flow cytometry analysis using Annexin V staining was performed to observe apoptosis (Fig. 4). Early apoptotic cells and late apoptotic or necrotic cells were represented in the Q3 and Q2 quadrants, respectively. The results showed that, compared with the control group, treatment with progressively increasing concentrations of Hcy significantly elevated the overall apoptotic cell rate, indicating that Hcy promotes apoptosis in KGN cells in a concentration-dependent manner.

### Integrated bioinformatic analysis of potential therapeutic targets for PCOS: PPI network construction, KEGG pathway, and GO enrichment analyses

**Fig. 5:**
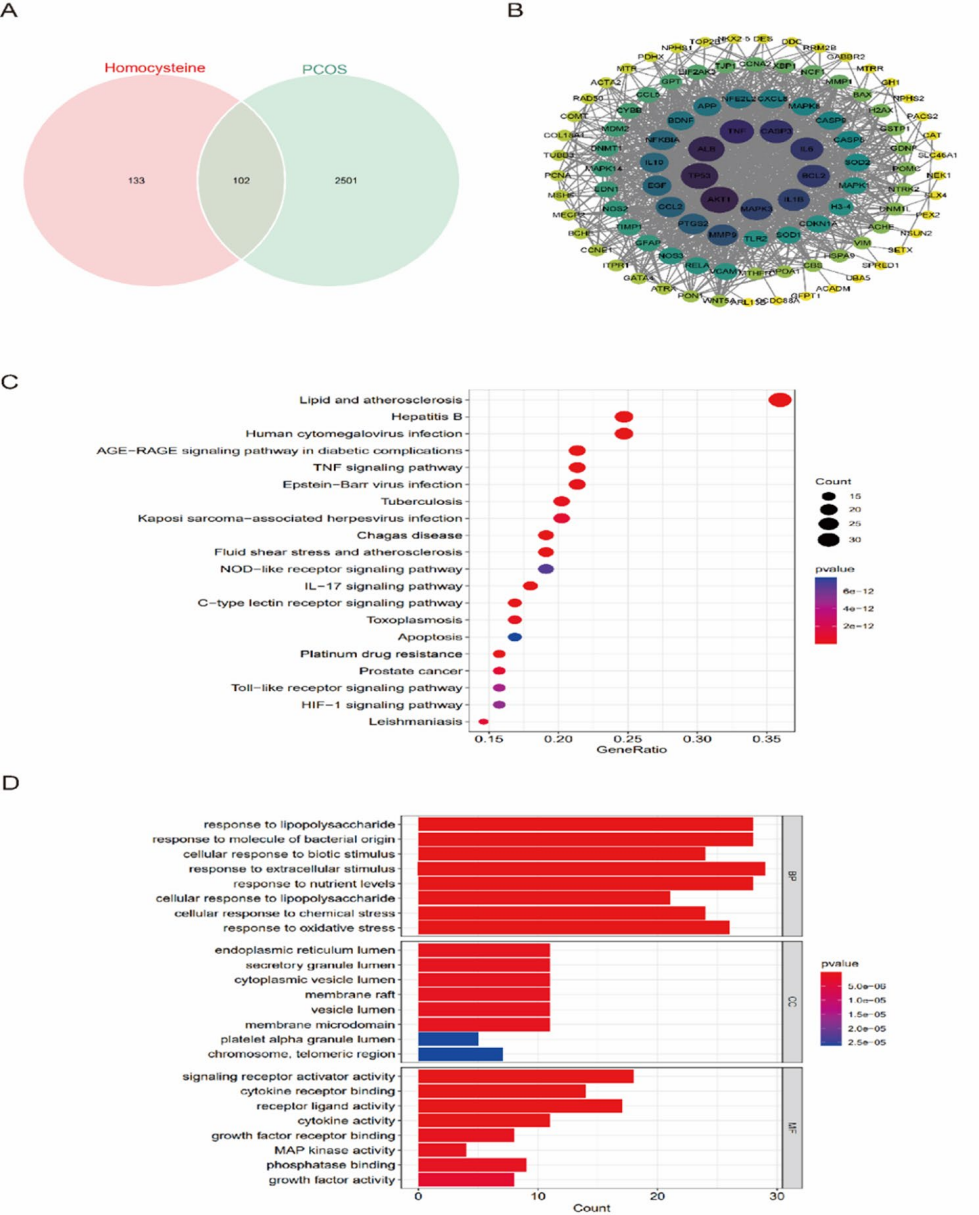
Venn diagram, PPI network of potential targets, KEGG, and GO enrichment analysis

Based on target identification, 102 potential Hcy-related therapeutic targets for PCOS were identified (Fig. 5A). A PPI network was constructed using the STRING database (Fig. 5B).

KEGG pathway enrichment analysis of these 102 targets identified the top 20 significantly enriched pathways (Fig. 5C), primarily involving lipid metabolism and atherosclerosis, hepatitis B, cytomegalovirus infection, the AGE-RAGE signaling pathway in diabetic complications, and the TNF signaling pathway.

GO enrichment analysis further revealed 293 biological process (BP) terms, 33 cellular component (CC) terms, and 56 molecular function (MF) terms (*p* < 0.05). The top 10 enriched GO categories, selected based on gene counts and statistical significance, are presented in Fig. 5D.

## Discussion

In this study, across the analyzed cohorts, there were no statistically significant differences in clinical pregnancy, miscarriage, or live birth rates. The role of Hcy in determining IVF/ICSI-ET outcomes remains controversial, with divergent findings reported in the literature. Evidence presented by Nafiye et al. aligns with the current observations, suggesting that although serum Hcy level may influence embryo number and quality, they do not significantly affect clinical pregnancy rates [33]. Similarly, Liu et al. reported no association between Hcy levels and oocyte maturation, embryo quality, or pregnancy outcomes [34]. In comparison, Ocal et al. showed a significant relationship between follicular fluid Hcy level and pregnancy outcomes, despite no correlation with oocyte quality. Lower Hcy levels were observed in the pregnancy group (9.6 ± 2.02 µmol/L) than in the non-pregnancy group (14.9 ± 2.93 µmol/L; *p* < 0.001), indicating its potential as a predictor of pregnancy failure [35]. Consistent with this, Berker et al. identified a significant correlation between follicular fluid Hcy levels and clinical pregnancy rates [36], while Boyama et al. further supported these findings by evaluating Hcy levels in embryo culture waste media [37]. Akamine et al. reported that follicular fluid Hcy levels below 4.9 µmol/mL were positively associated with improved pregnancy outcomes [38].

Previous studies have shown that, in patients with PCOS, endocrine abnormalities such as higher luteinizing hormone levels and hyperandrogenism after ovulation induction are frequently associated with implantation failure and increased miscarriage rates, which may be partially due to higher Hcy levels [39]. Elevated Hcy may disrupt endometrial receptivity by inducing oxidative stress in vascular endothelial cells, promoting platelet activation, impairing blood flow, stimulating vascular smooth muscle proliferation, and activating the UPR, ultimately leading to endothelial cell apoptosis. These changes may adversely affect embryo implantation and increase the risk of pregnancy loss [39]. At the molecular level, RNA sequencing of peripheral blood mononuclear cells from PCOS patients has identified 186 differentially expressed genes (69 upregulated and 117 downregulated genes). Among these, *AQP9*,* PROK2*,* S100A12*, and *TGM3* showed significant diagnostic potential for distinguishing PCOS patients from healthy controls [40]. Furthermore, a systematic review and meta-analysis found that polymorphisms in the vitamin D receptor (VDR) gene, including *ApaI*,* BsmI*,* Cdx2*, and *TaqI*, are associated with an increased risk of PCOS [41]. Higher Hcy levels have also been linked to adverse obstetric complications, including hypertensive disorders of pregnancy, intrauterine growth restriction, placental abruption, and preterm delivery, primarily through vascular dysfunction, abnormal uterine contractility, and oxidative stress [39, 42, 43]. Moreover, the interaction between Hcy and insulin resistance increases the risk of gestational diabetes mellitus [44].

Folate metabolism is a key regulatory pathway for Hcy homeostasis. Genetic variants in the *MTHFR* gene reduce enzymatic activity, impair folate metabolism, and elevate Hcy levels, increasing the risk of fetal neural tube defects and adverse obstetric outcomes. Due to the retrospective design of this study, prospective control of confounding variables (dietary intake and nutritional status) was not feasible, potentially influencing the results. To minimize potential bias, all clinical data, including Hcy measurements, were obtained from a standardized electronic medical record system, and both PCOS diagnostic criteria and IVF protocols were uniformly used. However, serum folate levels, vitamin B12 levels, and *MTHFR* gene polymorphisms were not examined or adjusted for, representing a limitation that should be addressed in future studies. The lack of association between Hcy levels and pregnancy outcomes observed in this study may be explained by several factors. First, no significant differences were observed among groups in endometrial thickness on the day of embryo transfer, nor in the number and quality of transferred embryos. Second, the timing of sample inclusion resulted in incomplete follow-up for some patients, potentially introducing bias and limiting the strength of conclusions about offspring outcomes.

Despite these findings, managing Hcy levels remains clinically important. Each 1 µmol/L increase in Hcy has been associated with a 1.4% reduction in the rate of high-quality embryos. Although this effect may be modest in a single treatment cycle, it may be clinically relevant in specific populations, such as patients with poor prognosis or recurrent implantation failure, particularly when cumulative outcomes are considered. Embryo morphology is an early developmental indicator and does not necessarily correlate directly with clinical pregnancy or live birth, both of which are influenced by multiple downstream factors, including endometrial receptivity and immune regulation. Therefore, the potential adverse effects of elevated Hcy on early embryonic development may be attenuated or masked during later stages of implantation and gestation. The results primarily indicate an association between Hcy levels and early embryonic developmental competence, whereas the impact of Hcy on final pregnancy outcomes remains inconclusive and needs further validation through large-scale prospective studies. The Hcy level range used in the in vitro experiments is in good agreement with a previous study investigating its effects on ovarian granulosa cell function, allowing for the observation of significant biological responses within a controlled timeframe [45].

The use of the KGN granulosa cell line is a limitation of this study. A comprehensive understanding of Hcy’s role in reproductive outcomes requires further investigation using endometrial cell models. Future studies should incorporate endometrial epithelial and stromal cells to directly evaluate the effects of Hcy on key markers of endometrial receptivity, including integrin αvβ3, leukemia inhibitory factor (LIF), and HOXA10, as well as its impact on cell adhesion and decidualization. Such approaches would provide direct mechanistic evidence linking Hcy dysregulation to impaired embryo implantation.

## Conclusion

Variations in Hcy levels were not associated with differences in clinical pregnancy outcomes across the study groups. However, higher Hcy levels were negatively correlated with the cleavage-stage high-quality embryo rate, with each 1 µmol/L increase associated with an approximate 1.4% decline. The results further indicated that Hcy exposure reduced KGN granulosa cell viability and promoted apoptosis. Network pharmacology analysis provided preliminary evidence supporting a potential mechanistic association between Hcy and PCOS.

## Data Availability

Data is provided within the manuscript or supplementary information files.

## References

[1] Ota K, et al. Effects of MTHFR C677T polymorphism on vitamin D, homocysteine and natural killer cell cytotoxicity in women with recurrent pregnancy losses. Hum Reprod. 2020;35(6):1276–87. 32478379 10.1093/humrep/deaa095

[2] Ostrakhovitch EA, Tabibzadeh S. Homocysteine and age-associated disorders. Ageing Res Rev. 2019;49:144–64. 30391754 10.1016/j.arr.2018.10.010

[3] Sørensen JT, et al. Molecular and biochemical investigations of patients with intermediate or severe hyperhomocysteinemia. Mol Genet Metab. 2016;117(3):344–50. 26750749 10.1016/j.ymgme.2015.12.010

[4] Gong T, et al. Serum homocysteine level and gestational diabetes mellitus: A meta-analysis. J Diabetes Investig. 2016;7(4):622–8. 27180921 10.1111/jdi.12460 PMC4931215

[5] Gaiday AN, et al. Effect of homocysteine on pregnancy: A systematic review. Chem Biol Interact. 2018;293:70–6. 30053452 10.1016/j.cbi.2018.07.021

[6] Akamine K, et al. Impact of the one-carbon metabolism on oocyte maturation, fertilization, embryo quality, and subsequent pregnancy. Reprod Med Biol. 2021;20(1):76–82. 33488286 10.1002/rmb2.12354 PMC7812474

[7] Revelli A, et al. Effects of Homocysteine Circulating Levels on Human Spontaneous Fertility and In Vitro Fertilization Outcomes: A Literature Review. Nutrients. 2025;17(20):3211. 10.3390/nu17203211 PMC12567194 41156464

[8] Wang L, et al. Effects of hyperhomocysteinemia on follicular development and oocytes quality. iScience. 2024;27(11):111241. 39563894 10.1016/j.isci.2024.111241 PMC11574796

[9] González F, et al. Oxidative Stress in Response to Saturated Fat Ingestion Is Linked to Insulin Resistance and Hyperandrogenism in Polycystic Ovary Syndrome. J Clin Endocrinol Metab. 2019;104(11):5360–71. 31298704 10.1210/jc.2019-00987 PMC6773460

[10] Rotterdam ESHRE. Revised 2003 consensus on diagnostic criteria and long-term health risks related to polycystic ovary syndrome. Fertil Steril. 2004;81(1):19–25. 10.1016/j.fertnstert.2003.10.004 14711538

[11] Dabravolski SA, et al. Mitochondrial Dysfunction and Chronic Inflammation in Polycystic Ovary Syndrome. Int J Mol Sci. 2021;22(8):3923. 10.3390/ijms22083923 PMC8070512 33920227

[12] Murri M, et al. Circulating markers of oxidative stress and polycystic ovary syndrome (PCOS): a systematic review and meta-analysis. Hum Reprod Update. 2013;19(3):268–88. 23303572 10.1093/humupd/dms059

[13] Ulloque-Badaracco JR, et al. Homocysteine, vitamin B12, and folate circulating levels in women with and without polycystic ovary syndrome: A systematic review and meta-analysis. Womens Health (Lond). 2024;20:17455057241279039. 39320480 10.1177/17455057241279039 PMC11437568

[14] Teede H, Deeks A, Moran L. Polycystic ovary syndrome: a complex condition with psychological, reproductive and metabolic manifestations that impacts on health across the lifespan. BMC Med. 2010;8:41. 20591140 10.1186/1741-7015-8-41 PMC2909929

[15] Jan M, et al. Molecular processes mediating hyperhomocysteinemia-induced metabolic reprogramming, redox regulation and growth inhibition in endothelial cells. Redox Biol. 2021;45:102018. 34140262 10.1016/j.redox.2021.102018 PMC8282538

[16] Lu H, et al. ZnT 9 Involvement in Estradiol-Modulated Zinc Homeostasis of the Human Follicular Microenvironment. Biol Trace Elem Res. 2024;202(5):1901–9. 37578601 10.1007/s12011-023-03804-y

[17] McRae C, et al. Metabolic profiling of follicular fluid and plasma from natural cycle in vitro fertilization patients–a pilot study. Fertil Steril. 2012;98(6):1449–e576. 22921074 10.1016/j.fertnstert.2012.07.1131

[18] Steegers-Theunissen RP, et al. Study on the presence of homocysteine in ovarian follicular fluid. Fertil Steril. 1993;60(6):1006–10. 8243678 10.1016/s0015-0282(16)56401-2

[19] Lin M, et al. Bisphenol A promotes autophagy in ovarian granulosa cells by inducing AMPK/mTOR/ULK1 signalling pathway. Environ Int. 2021;147:106298. 33387880 10.1016/j.envint.2020.106298

[20] Tan W, et al. MiR-93-5p promotes granulosa cell apoptosis and ferroptosis by the NF-kB signaling pathway in polycystic ovary syndrome. Front Immunol. 2022;13:967151. 36341347 10.3389/fimmu.2022.967151 PMC9626535

[21] Yu H, et al. Exposure to 6PPD-Q induces dysfunctions of ovarian granulosa cells: Its potential role in PCOS. J Hazard Mater. 2025;486:137037. 39764971 10.1016/j.jhazmat.2024.137037

[22] Ma LZ, et al. USP14 inhibition promotes DNA damage repair and represses ovarian granulosa cell senescence in premature ovarian insufficiency. J Transl Med. 2024;22(1):834. 39261935 10.1186/s12967-024-05636-3 PMC11389224

[23] Daina A, Michielin O, Zoete V. SwissTargetPrediction: updated data and new features for efficient prediction of protein targets of small molecules. Nucleic Acids Res. 2019;47(W1):W357–64. 31106366 10.1093/nar/gkz382 PMC6602486

[24] Yao ZJ, et al. TargetNet: a web service for predicting potential drug-target interaction profiling via multi-target SAR models. J Comput Aided Mol Des. 2016;30(5):413–24. 27167132 10.1007/s10822-016-9915-2

[25] Liu Z, et al. BATMAN-TCM: a Bioinformatics Analysis Tool for Molecular mechANism of Traditional Chinese Medicine. Sci Rep. 2016;6:21146. 26879404 10.1038/srep21146 PMC4754750

[26] Wang X, et al. PharmMapper 2017 update: a web server for potential drug target identification with a comprehensive target pharmacophore database. Nucleic Acids Res. 2017;45(W1):W356–60. 28472422 10.1093/nar/gkx374 PMC5793840

[27] Collaborators. The Universal Protein Knowledgebase in 2023. Nucleic Acids Res. 2023;51(D1):D523–31. 10.1093/nar/gkac1052 PMC9825514 36408920

[28] Amberger JS, et al. OMIM.org: Online Mendelian Inheritance in Man (OMIM^®^), an online catalog of human genes and genetic disorders. Nucleic Acids Res. 2015;43(Database issue):D789–98. 25428349 10.1093/nar/gku1205 PMC4383985

[29] Safran M, et al. GeneCards Version 3: the human gene integrator. Database (Oxford). 2010;2010:pbaq020. 10.1093/database/baq020 PMC2938269 20689021

[30] Wishart DS, et al. DrugBank 5.0: a major update to the DrugBank database for 2018. Nucleic Acids Res. 2018;46(D1):D1074–82. 29126136 10.1093/nar/gkx1037 PMC5753335

[31] Piñero J, et al. The DisGeNET knowledge platform for disease genomics: 2019 update. Nucleic Acids Res. 2020;48(D1):D845–55. 31680165 10.1093/nar/gkz1021 PMC7145631

[32] Yan H, et al. Exploring the mechanism of action of Yiyi Fuzi Baijiang powder in colorectal cancer based on network pharmacology and molecular docking studies. Biotechnol Genet Eng Rev. 2023;39(2):1107–27. 36735641 10.1080/02648725.2023.2167765

[33] Nafiye Y, et al. The effect of serum and intrafollicular insulin resistance parameters and homocysteine levels of nonobese, nonhyperandrogenemic polycystic ovary syndrome patients on in vitro fertilization outcome. Fertil Steril. 2010;93(6):1864–9. 10.1016/j.fertnstert.2008.12.024 19171332

[34] Liu L, et al. Association between serum homocysteine level and unexplained infertility in in vitro fertilization/intracytoplasmic sperm injection (IVF/ICSI): A retrospective, hospital-based, case-control study. J Clin Lab Anal. 2020;34(5):e23167. 10.1002/jcla.23167 PMC7246389 31876071

[35] Ocal P, et al. The association between homocysteine in the follicular fluid with embryo quality and pregnancy rate in assisted reproductive techniques. J Assist Reprod Genet. 2012;29(4):299–304. 10.1007/s10815-012-9709-y PMC3309985 22271234

[36] Berker B, et al. Homocysteine concentrations in follicular fluid are associated with poor oocyte and embryo qualities in polycystic ovary syndrome patients undergoing assisted reproduction. Hum Reprod. 2009;24(9):2293–302. 10.1093/humrep/dep069 19443458

[37] Boyama B, et al. Homocysteine in embryo culture media as a predictor of pregnancy outcome. Assist reproductive Technol. 2016;32(3):193–5. 10.3109/09513590.2015.1102877 26806445

[38] Akamine K, et al. Impact of the one-carbon metabolism on oocyte maturation, fertilization, embryo quality, and subsequent pregnancy. 2021;20(1):76–82. 10.1002/rmb2.12354 PMC7812474 33488286

[39] Brauer P, B.J.C.p.d. Tierney, Consequences of elevated homocysteine during embryonic development and possible modes of action. 2004;10(22):2719-32. 10.2174/1381612043383692 15320738

[40] Heidarzadehpilehrood R, et al. Identifying Genetic Profiles in Peripheral Blood Mononuclear Cells in Women with Polycystic Ovary Syndrome: An Observational Case-Control Study. Arch Med Res. 2025;56(3):103129. 39647252 10.1016/j.arcmed.2024.103129

[41] Heidarzadehpilehrood R, Hamid HA, Pirhoushiaran M. Vitamin D receptor (VDR) gene polymorphisms and risk for polycystic ovary syndrome and infertility: An updated systematic review and meta-analysis. Metabol Open. 2025;25:100343. 39866289 10.1016/j.metop.2024.100343 PMC11764755

[42] Gaiday A, et al. Effect of homocysteine on pregnancy: A systematic review. 2018;293:70–76. 10.1016/j.cbi.2018.07.021 30053452

[43] Dai C, et al. A Novel Review of Homocysteine and Pregnancy Complications. Biomed Res Int. 2021;2021:6652231. 10.1155/2021/6652231 PMC8121575 34036101

[44] Zheng Y, et al. Homocysteine level and gestational diabetes mellitus: a systematic review and meta-analysis. Gynecol Endocrinol. 2021;37(11):987–94. 10.1080/09513590.2021.1967314 34409893

[45] Shi Q, Liu R, Chen L. Ferroptosis inhibitor ferrostatin–1 alleviates homocysteine–induced ovarian granulosa cell injury by regulating TET activity and DNA methylation. Mol Med Rep. 2022;25(4). 10.3892/mmr.2022.12645 PMC8867468 35169856

